# Involving consumers and the community in the development of a diagnostic instrument for fetal alcohol spectrum disorders in Australia

**DOI:** 10.1186/1478-4505-11-26

**Published:** 2013-07-30

**Authors:** Heather M Jones, Anne McKenzie, Sue Miers, Elizabeth Russell, Rochelle E Watkins, Janet M Payne, Lorian Hayes, Maureen Carter, Heather D’Antoine, Jane Latimer, Amanda Wilkins, Raewyn C Mutch, Lucinda Burns, James P Fitzpatrick, Jane Halliday, Colleen M O’Leary, Elizabeth Peadon, Elizabeth J Elliott, Carol Bower

**Affiliations:** 1 Telethon Institute for Child Health Research, Centre for Child Health Research, The University of Western Australia, PO Box 855, West Perth, Western Australia 6872, Australia; 2 National Organisation for Fetal Alcohol Syndrome and Related Disorders, PO Box 206, Normanville, South Australia 5204, Australia; 3 Russell Family Fetal Alcohol Disorders Association, PO Box 6795, Cairns, Queensland 4870, Australia; 4 Centre for Chronic Disease, School of Medicine, University of Queensland, 288 Herston Road, Herston, Brisbane, Queensland 4006, Australia; 5 Nindilingarri Cultural Health Services, PO Box 59, Fitzroy Crossing, Western Australia 6765, Australia; 6 Menzies School of Health Research, Charles Darwin University, PO Box 41096, Casuarina, Northern Territory 0811, Australia; 7 The George Institute for Global Health, PO Box M201, Missenden Road, Sydney, New South Wales 2050, Australia; 8 Department of Health Western Australia, Child and Adolescent Health Service, PO Box S1296, Perth, Western Australia 6845, Australia; 9 National Drug and Alcohol Research Centre, University of New South Wales, Sydney, New South Wales 2052, Australia; 10 Public Health Genetics, Genetic Disorders, Murdoch Childrens Research Institute, Royal Children’s Hospital, Flemington Road, Parkville, Victoria 3052, Australia; 11 Department of Paediatrics, University of Melbourne, Royal Children’s Hospital, 50 Flemington Road, Parkville, Victoria 3052, Australia; 12 Centre for Population Health Research, Curtin University, GPO Box U1987, Perth, Western Australia 6845, Australia; 13 Discipline of Paediatrics and Child Health, Sydney Medical School, University of Sydney, Locked Bag 4001, Westmead, New South Wales 2145, Australia; 14 The Children’s Hospital at Westmead, Locked Bag 4001, Westmead, New South Wales 2145, Australia

**Keywords:** Consumer participation, Fetal alcohol spectrum disorder, Research

## Abstract

**Background:**

Australia’s commitment to consumer and community participation in health and medical research has grown over the past decade. Participatory research models of engagement are the most empowering for consumers.

**Methods:**

As part of a project to develop a diagnostic instrument for fetal alcohol spectrum disorders (FASD) in Australia (FASD Project), the Australian FASD Collaboration (Collaboration), including a consumer advocate and two consumer representatives, was established. On completion of the FASD Project an on-line survey of Collaboration members was conducted to assess their views on consumer involvement. Women in the community were also invited to participate in Community Conversations to discuss real life situations regarding communications with health professionals about alcohol and pregnancy. Community Conversation feedback was analysed qualitatively and attendees were surveyed about their views of the Community Conversation process.

**Results:**

The on-line survey was completed by 12 members of the Collaboration (71%). Consumer and community participation was considered important and essential, worked well, and was integral to the success of the project. The 32 women attending the Community Conversations generated 500 statements that made reference to prevention, how information and messages are delivered, and appropriate support for women. Nearly all the attendees at the Community Conversations (93%) believed that they had an opportunity to put forward their ideas and 96% viewed the Community Conversations as a positive experience.

**Conclusions:**

The successful involvement of consumers and the community in the FASD Project can be attributed to active consumer and community participation, which included continued involvement throughout the project, funding of participation activities, and an understanding of the various contributions by the Collaboration members.

## Background

Australia is progressively moving towards actively encouraging health researchers to engage with consumer and community members. In 1992, Jenny Macklin, the Director of the National Health Strategy, referred to participation as a democratic right reinforced by government legislation. This approach to participation is centred on three factors: 1) public participation at a range of levels; 2) focus on consumers and communities, rather than providers and funder interests; and 3) an open system encompassing information, public accountability, and transparent and conspicuous decision-making [1]. The 1998 Wills Review into health and medical research in Australia [2] recommended that 1) researchers, health care providers and consumers join together in identifying and ranking priority areas using an accepted framework; 2) consumers who take part in research should be told about the outcomes; and 3) researchers should disseminate information about the role, benefits, results, and ethics of new research and its consequences for the community.

The National Health and Medical Research Council (NHMRC) National Statement on Ethical Conduct in Human Research also refers to the need for research that is of “benefit to the community” [3]. The Australian Code for the Responsible Conduct of Research (the Code) jointly developed by the NHMRC, the Australian Research Council and Universities Australia, defines research as “original investigation to gain knowledge, understanding and insight” [4]. The Code bases this definition on the Research Assessment Exercise for universities in the United Kingdom (UK), which refers to “work of direct relevance to the needs of commerce, industry and to the public and voluntary sectors” [5].

Consumer and community participation in Australian health and medical research has been advanced by consumer organisations such as Consumers Health Forum of Australia (CHF) [6], Cancer Council New South Wales (NSW) [7], and the National Association of People Living with HIV/AIDS [8]. Through a partnership between the NHMRC and CHF, the Statement on Consumer and Community Participation in Health and Medical Research (the Statement) was published in 2002. Their shared vision was that “consumers and researchers would work together in partnerships based on understanding, respect and shared commitment to research that will improve the health of human kind” [9]. Consumers and researchers formed an alliance to develop a Model Framework for Consumer and Community Participation in Health and Medical Research (Model Framework) [10]. The Model Framework was based on the objectives outlined in the Statement. In order to comply with the Statement, researchers applying for NHMRC project grant funding are required to complete a section on Consumer and Community Participation [11].

Underpinned by the principles in the Statement, a joint consumer and community engagement program began at the Telethon Institute for Child Health Research (Telethon Institute) and the School of Population Health at The University of Western Australia. This initial program has been extended and enriched to become the Consumer and Community Participation Program (Participation Program) [12]. This included a series of workshops facilitated by the consumer advocate and attended by researchers from both organisations, to discuss ways to help researchers think about increasing involvement of consumers and the community in research.

It is important that participation in health and medical research by consumers and the community is “active” – researchers, consumers and community members working together to make decisions about the research. Participation is having influence over a decision, having a say in what happens, contributing to policy development or being part of the process [13-15]. Whereas consultation is the more passive form of participation where consumers are asked their views but are not involved in deciding or doing the research [15]. Various models of consumer and community participation have been developed to provide guidance to researchers. These include ladder models [16,17], continuums or steps, each using different methods to describe levels of participation [18], and classifications of the type of involvement [19-21]. Action and participatory models of research are referred to in the literature as the most empowering for consumers, with collaboration favoured for sharing power and getting the balance between what is being researched and what will benefit the community [22]. In 2009 the Victorian Department of Health introduced the Strategic Direction 2010–2013 “Doing it with us not for us” which defines participation as occurring “… when consumers, carers and community members are meaningfully involved in decision-making about health policy and planning, care and treatment, and the wellbeing of themselves and the community. It is about having your say, thinking about why you believe in your views and ideas of others. In working together, decisions may include a range of perspectives” [23].

Consumers and community members provide a different perspective to health professionals and researchers based on their lived experience, and can ground the research on what is important for patients and their families, and communities. They know first-hand the areas of concern and what are the unmet needs for diagnosis, service delivery and care. Consumer perspectives also complement the perspectives of clinicians and researchers by providing a more holistic interpretation of health [22] and they are able to use a “personal lens” [24]. Telford et al. refer to “widening the scope of health research” and mention that researchers may be focused on understanding while consumers are more interested in implementing research findings [19]. The Participation Program has developed a method of seeking input from the community about gaps in current research projects and their priorities for future research. Adapted from the Cancer Council NSW, the method has developed at the Telethon Institute into Community Conversations. Since 2009 over 20 Community Conversations have been held on a wide range of topics as part of the Participation Program’s activities.

Between August 2010 and May 2012 we undertook the FASD Project to develop a diagnostic instrument for FASD in Australia and established the Collaboration to guide the project. The FASD Project consisted of a systematic literature review; Community Conversations; a Delphi study; a consensus development workshop; and diagnostic and consumer subgroup meetings. We report here the consumer and community contributions to the FASD Project; findings from the Delphi study component have been reported separately [25-27].

## Methods

Consistent with the Telethon Institute’s policy of consumer and community participation and the ethos of the Participation Program, we sought advice from the consumer advocate in the developmental stages of the FASD Project, and based on this advice we applied the principles expressed in the Statement [9] and the stages of the research cycle in the Model Framework [10].

### Australian FASD collaboration

In addition to the Consumer Advocate Anne McKenzie, invitations were extended to non-government organisations representing the interests of parents, carers and others involved in or affected by FASD to nominate a representative to the Collaboration. Sue Miers AM, National Organisation for Fetal Alcohol Syndrome and Related Disorders (NOFASARD), and Elizabeth Russell, Russell Family Fetal Alcohol Disorders Association, agreed to be members of the Collaboration. Other members of the Collaboration were Winthrop Research Professor Carol Bower, Professor Elizabeth Elliott AM, Dr Lucinda Burns, Maureen Carter, Heather D’Antoine, Dr James Fitzpatrick, Associate Professor Jane Halliday, Lorian Hayes, Associate Professor Jane Latimer, Dr Raewyn Mutch, Dr Colleen O’Leary, Dr Janet Payne, Dr Elizabeth Peadon, Dr Amanda Wilkins, Dr Rochelle Watkins, and Heather Jones. The role of the Collaboration was to provide high-level expertise, advice, expert opinion, and peer-review for the project. The FASD Project budget included honoraria for Collaboration members and travel and accommodation expenses to attend the consensus development workshop.

Collaboration members met on 14 occasions via teleconference during the 22-month FASD Project and participated in a two-day face-to-face consensus development workshop. At the conclusion of the FASD Project, members of the Collaboration were asked to complete a 20-question on-line evaluation survey. Based on the work of Payne et al., four open-ended questions specific to consumer and community participation in the FASD Project were included in the survey [28] (Table 1).

**Table 1:** FASD Collaboration evaluation open-ended questions on the impact of consumer and community participation in the FASD Project

**Open-ended questions**	**FASD Collaboration n = 17**
	**Returned surveys n = 12**
	Responded
*What impact do you think consumer and community participation made to the FASD Project?*	11 (67%)
*What was learned about consumer and community participation that worked well?*	7 (41%)
*What was learned about consumer and community participation that did not work well?*	6 (35%)
*What changes to consumer and community participation can you suggest for future projects?*	6 (35%)

### Systematic literature review

In the planning process, Collaboration members identified their involvement in FASD research and expected contributions to the FASD Project. Members with expertise in reviewing literature and extracting data were invited to participate in the literature review.

### Community conversations

The Collaboration decided to conduct two Community Conversations with community members to obtain wider community input for the FASD Project; one in Perth, Western Australia, and one in Cairns, Queensland. Invitations to participate in a Community Conversation seminar on alcohol and pregnancy were placed on websites and emailed to a network of consumer and community organisations, reference groups, and women living in Perth and Cairns known to members of the Collaboration. Consumer organisations contacted were broad and not restricted to child health issues. We acknowledge that the participants are unlikely to be representative of the broader population, particularly with respect to people who do not have access to electronic communication or high-risk populations. A modified version of the world café concept [29] was used to engage women in conversations that reflected real life situations about alcohol and pregnancy and communication with health professionals. Questions for use at the Perth Community Conversation were developed by the consumer advocate and consumer representative members of the Collaboration, one of the lead investigators, and the FASD Project manager (Table 2) and, following the Perth meeting, were altered slightly for use in Cairns (Table 3). Using the world café process and led by table facilitators, participants in each small group discussed the question around their table and wrote their individual statements on sticky notes and placed them on a large sheet of paper. At regular intervals (30 minutes) participants moved to a new table with the table facilitator remaining in place. At the end of the process the main ideas were summarised by the table facilitators and follow-up possibilities were discussed by the whole group. Women attending the Community Conversations were provided with an honorarium to cover out-of-pocket expenses such as parking or public transport and refreshments were provided.

**Table 2:** Questions used at the Perth Community Conversation

**Background provided to participants**	**Questions**
Research indicates that health professionals have an important role to play in the prevention of prenatal alcohol exposure. Women expect health professionals to ask and advise them about alcohol during pregnancy. However, the majority of health professionals in Western Australia do not routinely ask pregnant women about alcohol use or provide them with information about the consequences of alcohol use in pregnancy.	a) *If you were pregnant, what would you want your health professional to say to you to about alcohol?*
	*b) How would you want the health professional to raise it with you? Are there ways of asking that might work better for particular groups of women or that account for cultural sensitivities?*
	*c) What information would you want a health professional to give you?*
	*d) Would it be any different if the information came from a midwife, community health nurse, GP or an obstetrician?*
Currently, information is collected by midwives on all mothers and newborn babies. There is information on the baby such as weight, length and head circumference; labour and delivery details; and details on the mother such as age, height, marital status, ethnic origin, previous pregnancies and smoking during pregnancy. This information is recorded on the midwives’ Notification of Birth Form.	a) *If you had just given birth, would you agree to answer a question (or questions?) about your alcohol use during pregnancy?*
	*b) What do you think is the best way for a health professional to ask this question? Are there ways of asking that might work better for particular groups of women or that account for cultural sensitivities?*
Research has shown that there is confusion about ‘what are a few drinks’ and the alcohol content of various drinks. Therefore, just asking if you have consumed any alcohol during pregnancy does not provide sufficient information to health professionals. You may refer the participants to the ‘Standard Drinks’ Guides in their handouts.	*How would you feel if you were pregnant and asked to provide more detail about your alcohol use? This information could include:*
	*a) When during the nine months of your pregnancy did you drink alcohol (months 1–3, months 4–6, months 7–9)?*
	*b) How much alcohol did you drink at each occasion (for example 3 full strength beers, 1 glass of wine)?*
	*c) How frequent were those occasions when you drank alcohol (for example three times a day, daily, weekly, etc.)?*
	*d) What do you think is the best way for a health professional to ask these questions? Are there ways of asking that might work better for particular groups of women or that account for cultural sensitivities?*
Delayed development, low IQ and learning difficulties in children can be caused by a range of factors, including prenatal alcohol exposure.	a) *If you had a child with delayed development, low IQ or learning difficulties, would you agree to answer questions about your alcohol use during pregnancy?*
	*b) What do you think is the best way for a health professional to ask these questions? Are there ways of asking that might work better for particular groups of women or that account for cultural sensitivities?*

**Table 3:** Questions used at the Cairns Community Conversation

**Background**	**Questions**
Research indicates that health professionals have an important role to play in the prevention of prenatal alcohol exposure. Women expect health professionals to ask and advise them about alcohol during pregnancy. However, the majority of health professionals do not routinely ask pregnant women about alcohol use or provide them with information about the consequences of alcohol use in pregnancy.	*If you were pregnant, what would you want your health professional to say or provide to you about alcohol use and its potential harm?*
Research has shown that there is confusion about ‘what are a few drinks’ and the alcohol content of various drinks. Therefore, just asking if you have consumed any alcohol during pregnancy does not provide sufficient information to health professionals. You may refer the participants to the ‘Standard Drinks’ Guides in their handouts.	*How would you feel answering questions about your alcohol use either during pregnancy or straight after giving birth? These questions might include:*
Currently information is collected by midwives on all mothers and newborn babies. There is information on the baby such as weight, length and head circumference; labour and delivery details; and details on the mother such as age, height, marital status, ethnic origin, previous pregnancies and smoking during pregnancy. This information is recorded on the midwives’ Notification of Birth Form.	*a) When during the nine months of your pregnancy did you drink alcohol (months 1–3, months 4–6, months 7–9)?*
	*b) How much alcohol did you drink at each occasion (for example 3 full strength beers, 1 glass of wine)?*
	*c) How frequent were those occasions when you drank alcohol (for example three times a day, daily, weekly, etc.)?*
You are talking to a health professional who is assessing your child who has delayed development, low IQ and/or learning difficulties. Delayed development, low IQ and/or learning difficulties in children can be caused by a range of factors. The health professional will need to ask many questions about your pregnancy, family health history and information about your child.	*If you had a child with delayed development or learning difficulties, how would you feel about being asked questions by the health professional about your alcohol use during pregnancy?*
	*Are there any other issues that should be taken into consideration or discussed in relation to alcohol and pregnancy?*

The Community Conversations were facilitated by the consumer advocate and conducted in an open and friendly but safe environment. The consumer members of the Collaboration gave a presentation about living with a child with FASD and another Collaboration member presented on the clinical aspects of FASD. Following the philosophy of the Community Conversation and world café process, conversations were not recorded and field notes were not taken. The 500 participant statements were entered into a spreadsheet using word processing software and coding was performed by two authors using hardcopy records. Using qualitative content analysis [30,31] the statements were coded inductively based on the words used and underlying meaning of the statements. A second stage of analysis was conducted on the theme statements to identify key issues that were: 1) specific to the FASD Project; 2) required action on the completion of the FASD Project; and 3) related to alcohol and pregnancy and FASD but were not specific to the FASD Project. The women who attended the Community Conversations were also asked to complete an evaluation form. A total of eight Likert statements and five open-ended questions were included in the evaluation (Table 4). Agreement with the Likert statements was assessed on a 6-point scale (from 1 = positive to 6 = negative).

**Table 4:** Community Conversation evaluation

**Likert statements**	**Responses (very positive/positive)**	**Total number of responses**
1. The Community Conversation was informative	24 (83%)	29
2. The Community Conversation was useful	22 (76%)	29
3. The Community Conversation was participative	27 (96%)	28
4. Did the Community Conversation meet your expectations?	27 (90%)	30
5. Did the Community Conversation cover most areas that were important to you?	24 (80%)	30
6. Did the presentation on current research provide enough information?	21 (70%)	30
7. How well were your questions answered?	22 (73%)	30
8. Did you have an opportunity to put forward your ideas/priorities for research?	28 (93%)	30
**Summary of responses to open-ended questions**		
9. Is there anything else you would like to add?	Doctors need to give correct information that ‘no alcohol is safe when pregnant’	
	What we said was valued	
	Need to provide information to high school students	
	More information on FASD research and issues surrounding diagnosis	
	Health professionals have different perspective to community members	
	Great to have access to up-to-date sharing of information and resources	
	Inspiring presentations by parents living with children with a FASD	
10. The best thing about the Community Conversation was:	Very informative	
	Our voices and points of view were heard	
	Hearing different opinions	
11. The worst thing about the Community Conversation was:	Questions repetitive and not deep enough	
	More time for discussion	
	Not enough pregnant women or Aboriginal women	
12. Do you have any suggestions about how we might improve future Community Conversations?	Longer time	
	Questions sent to participants prior to Community Conversation	
	Different process to world café	
	More time for questions to speakers	
13. Would you be interested in attending future Community Conversations on other research areas at the Telethon Institute?	Yes	26
	No	1
	Maybe	2

### Delphi study

Data from the systematic literature review and the key issues from the Community Conversations were reviewed by the Collaboration to develop questions for the Delphi survey for health professionals. The modified Delphi process is described in Watkins et al. [27]. Members of the Collaboration were asked to identify individuals known to have expertise or experience in the screening or diagnosis of FASD for recruitment to the Delphi study panel.

### Consensus development workshop

All members of the Collaboration were invited to participate in the two-day workshop to review the evidence from the systematic review of the literature, feedback from the Community Conversations and results from the Delphi study, and develop the diagnostic instrument for FASD in Australia. The workshop methods are described elsewhere (Watkins RE, Elliott EJ, Wilkins A, Mutch RC, Fitzpatrick JP, Payne JM, O’Leary CM, Jones HM, Latimer J, Hayes L, Halliday J, D’Antoine H, Miers S, Russell E, Burns L, McKenzie A, Peadon E, Carter M, Bower C: Recommendations from a consensus development workshop on the diagnosis of fetal alcohol spectrum disorders in Australia, submitted).

### Diagnostic and consumer subgroups

Based on a decision from the consensus development workshop, a six-member clinician diagnostic subgroup and a three-member consumer subgroup of the Collaboration were formed. The consumer subgroup (the consumer advocate and the representatives from NOFASARD and RFFADA) and Project manager met via teleconference to review the consensus development workshop and Community Conversation outcomes to develop three consumer resources (an information sheet for clinicians, an information sheet for parents and carers, and a consent form) for inclusion in the guidelines to accompany the diagnostic instrument.

### Ethics

The FASD Project was approved by The University of Western Australia Human Research Ethics Committee and the Western Australian Aboriginal Health Information and Ethics Committee.

## Results

### Australian FASD collaboration

Consumer members of the Collaboration were involved in all meetings and attended the consensus development workshop. An on-line anonymous survey distributed at the end of the project was completed by 12 of the 17 members of the Collaboration (71%). Not all members returning the survey completed all four questions pertaining to consumer and community participation (Table 1). A total of 30 statements were submitted in response to questions on consumer and community participation in the FASD Project. Overall, Collaboration members thought that consumer and community participation was important and essential, worked well, and was integral to the success of the project. In particular, members commented on two aspects of the impact of consumer and community participation in the FASD Project: community voice and inclusiveness.

Eight members made comments regarding the views, experience and knowledge of consumers, and the community “voice”. These included “*… bring a different perspective to health professionals and researchers*”, “*… a reminder of the world outside research*” and “*…they bring balance*”. The participation by consumers and the community enhances the outcomes and makes the research relevant by identifying what would work for affected children and their families with one member noting “*What’s done in the ‘lab’ must be able to produce some results for people at the grass roots*”.

Collaboration members also commented that the consumer representatives were “*equal contributors and their eagerness and commitment was unquestionable*”, and remarked that “*The group was comfortable with us there*”, “*…we were treated respectfully*”, “*we seemed to fit in with the academics*”, and there was “*a lot of good will*”*.* This statement from a Collaboration member summarises the changing face on consumer and community participation in research: “*Originally I was somewhat doubtful about the importance of having consumer voices included in a project about diagnosis. However, I think these voices were extremely important in grounding the process of diagnosis and ensuring that the recommendation made in the final report, and were considered in light of what would work best for affected children and their families. Additionally I think the consumers will be important advocates of the diagnostic tool that has been developed during this process*”*.* However, one respondent commented “*some consumers are unable to look generally at a problem without personalising it and this may lead to a misunderstanding that their opinions are not adequately valued*”*.*

### Community conversations

Participants were women of child-bearing age and included women with and without children and some who were pregnant. We did not collect demographic details from individual participants. A total of 500 statements were collected from the 32 women who attended the two Community Conversations (25 in Perth, 7 in Cairns). Table 5 lists the 13 themes and summarises the statements under each theme. The selected statements that follow are indicative of the issues raised by attendees. Many attendees noted that prevention and need to take the message to the whole community, including men is a priority. One participant stated “*Educate women so it isn’t just another thing she can’t do – put it on TV, everyone sees it*” and another commented “*Not just mother – whole family system*”. Other attendees also referred to the use of television and social media with statements: “*More emphasis on national recognition of FAS rather than putting sole responsibility on the GPs to deliver education on alcohol consumption, e.g., more info via TV, radio, etc.*” and “*Using websites, i.e., headspace, myspace, facebook, bebo*”. Participants also highlighted young people’s education saying “*Educate young people about the effects of drinking on babies. Focus on the positives of how to have a healthy baby*”.

**Table 5:** Summary of participant statements

**Theme (number of statements)**	**Summary of participant statements**
**Information to public (5)**	• Prevention is the priority and there should be a national campaign – TV, radio, posters, coasters, fridge magnets, social media and information in pubs, clubs, bars, behind toilet doors, Centrelink, Medicare, doctors and clinic waiting rooms, public transport
	• Need for warning labels on alcoholic beverages
**Information to women (10)**	• Use of visual aids to explain how alcohol actually affects the baby
	• Messages from health professionals should be consistent and be honest that there is no known safe limit for drinking alcohol during pregnancy
	• Awareness that even though FASDs are not curable, the correct diagnosis can help with strategies for the child and family
**How to ask questions about alcohol use (15)**	• Make the question about alcohol use part of a standard set of questions that are asked in the context of diet and lifestyle for all pregnant women. Put an equal emphasis on alcohol as other substances such as tobacco or drugs
	• Acknowledge that there is no single way of asking that will please everyone
	• Questions should be simple, clear and easy to understand for all races/classes within society and not a lecture or interrogation. Should also recognise cultural sensitivities and that nodding the head does not always mean ‘yes’, I agree
	• Explain how alcohol affects the baby and how it crosses the placenta – everything the mother drinks reaches the baby and the baby will be drunk with her
	• Health professionals should be non-judgemental and prepared to deal with feelings of defensiveness, fear, guilt, shame, panic and the ‘what have I done’ questions. Need to focus on the future not on the past
**Language (4)**	• Simplify the terminology, consider language barriers and the use of visual aids
**Timing (7)**	• Information to women and community on alcohol use in pregnancy so women are better informed before they get pregnant
	• Health professionals should talk about alcohol use before women become pregnant and at regular visits to GP by young women and women who may be contemplating becoming pregnant and build up a relationship that will continue into pregnancy
	• Should be part of a routine set of questions asked by the midwife of all women at birth – should not be in an admission pack questionnaire
**Feelings (16)**	• Defensive, confronted, concerned, ashamed, anxious and offended
	• A feeling of guilt and shame, or doing something wrong and wanting to know why the health professional is asking the question about alcohol consumption
	• Stereotyped by race/ethnicity
**Counselling support (6)**	• Health professionals need to know where and how to refer women and/or family members to support and counselling services
	• Women need support, not judgement or to be made to feel guilty. Health professionals should be mindful of mental health issues
**Family community (5)**	• Information about alcohol use in pregnancy should be available to the whole community, not just women. Families (including men) need to help support other women/men who might be thinking about having a baby. This support is important. It is hard when communities/friends are all drinking and the pregnant woman isn’t accepted or doesn’t feel part of the group
	• Questions about alcohol use should be asked in private and not in front of partners or family members
	• This is about the child and their difficulties, not about their culture
**Health professionals (9)**	• Important for health professionals to build relationships. Women prefer information coming from a child health nurse, midwife or female doctor as they take more time and seem more caring. Building trust between health professionals and women is important as there are shame factors associated with how much a woman has been drinking
	• Research and women’s feedback is that health professionals are not providing information to women or they are giving mixed messages about alcohol use in pregnancy
	• Health professionals should ask a woman what she knows about alcohol and pregnancy and ask if she would like to talk about this or would like to take some information away to read. Explain why these questions are being asked and that you are asking all pregnant women
**Health professional training (6)**	• All health professionals need to be trained in communicating with women about alcohol and pregnancy in a manner that is non-judgemental, language that is easy to understand and that is culturally sensitive
	• Training should commence at university with additional information as part of continuing professional development. Training should include information on what is a standard drink, risk factors, how to recognise FASD, diagnosis, and what difficulties a person with a FASD and their family will face in life
**Resources (4)**	• Preference for visual aids to help explain how alcohol gets to the baby and how it can affect the baby
	• Resources and information should be culturally appropriate and widely available in urban, regional and remote communities
**Schools (4)**	• Information on alcohol use on pregnancy should be part of the drug and alcohol health education curriculum for 12 – 16 year olds and not as a stand-alone subject and should focus on the effects of drinking alcohol on the developing baby and the positives of how to have a healthy baby
**General (14)**	• FASD is not curable – it’s for life
	• Rename FASD as it just points the finger at the mother and labels the child. The name should represent the symptoms not the cause
	• Establish a register of children with a FASD
	• Mandatory reporting of FASD. However, some women said pregnant women may be scared of mandatory reporting and fearful that they would be reported to the police and/or the Department for Child Protection
	• Parents/guardians should be asked if they want to proceed with screening, i.e., provide informed consent
	• A screening and diagnostic instrument must be appropriate for all Australian children, suitable for different ages and must provide a guide to referral pathways to appropriate health professionals
	• Sharing of information and resources and networking through a website and conferences
	• FASD should be on the agenda at community events and medical conferences

Community Conversation participants also referred to how information and messages are delivered to women. Participants supported the use of a standard set of questions for all women, with one attendee stating “*Health professionals can tell you it’s a standard question they ask everyone so you don’t think you are being singled out. Also only have someone with people skills to ask the question (not someone who will look horrified if you say yes)*”*.* Of particular importance was how to ask the question about alcohol consumption in pregnancy, the language used and training for health professionals to ask the question. Women wanted questions asked “*In a non-accusatory manner. People will clam up (go into denial) if confronted in a way that makes them feel to blame for their child’s condition*”*.* Women also commented that “*The health professional should be confident in asking the questions – not hiding behind language*” and that health professionals should “*Be aware of language issues – break down big words so people understand*”.

It was important to women attending the Community Conversations that health professionals recognise the feelings and anxieties when asking women about alcohol use in pregnancy. These concerns were reflected in statements such as “*Embarrassed (shame factor) ‘I am not a drunk’*” and “*Fear, guilt, panic – what irreversible decision have I made?*” While another participant stated “*Should be made to feel proud if you don’t drink*”*.* One woman commented “*Ask but at the same time offer solutions. Hard to offer up information if you are feeling there’s nothing being given in return, i.e., this information will help people in the future but it won’t necessarily help your situation*”.

Several attendees expressed concern about the need for cultural sensitivities with statements such as “*Intergenerational trauma and legislation for Aboriginal people*” and “*Assumption about culture that comes across as patronising*”. However, another participant stated “*This is about the child and their difficulties, not about their culture*”.

Some statements reflected concern about the name “Fetal Alcohol Spectrum Disorder” and that FASD is not curable. One participant stated “*Rename the syndrome coz FASD just points the finger at the mother, whereas some generic name like learning-blah-blah disorder doesn’t let others know the mum was at ‘fault’*”*.*

The majority of women attending gave positive or very positive responses to the evaluation questions about the Community Conversation (Table 4). Participants responded positively to the presentations from two consumer members of the Collaboration.

### Delphi study

Of the 220 health professionals invited to participate, 28 were recruited by consumer representative members of the Collaboration and women attending the Community Conversations. One of the key issues evolving from the Community Conversations was that a routine question about alcohol use should be asked of all pregnant women. This was included in the survey as follows “Alcohol exposure should be assessed alongside other lifestyle factors including diet, physical exercise and smoking”*.* Of the 103 health professionals who completed the survey, 92% agreed with this statement [27].

### Consensus development workshop

All consumer members of the Collaboration attended the workshop. Workshop participants identified a number of general principles to be included in the design of an instrument for the diagnosis of FASD in Australia. These principles are described elsewhere (Watkins RE, Elliott EJ, Wilkins A, Mutch RC, Fitzpatrick JP, Payne JM, O’Leary CM, Jones HM, Latimer J, Hayes L, Halliday J, D’Antoine H, Miers S, Russell E, Burns L, McKenzie A, Peadon E, Carter M, Bower C: Recommendations from a consensus development workshop on the diagnosis of fetal alcohol spectrum disorders in Australia, submitted) and are consistent with statements from the Community Conversations such as the requirement for training of health professionals; suitability of the diagnostic instrument for use across the lifespan; availability of support for the individual and family; and that parents, carers and individuals should be informed about the diagnostic process and provided with access to resources. It was also recommended that informed consent should be obtained and recorded prior to the communication of diagnostic findings to third parties such as General Practitioners or teachers.

### Consumer subgroup

The three-member consumer subgroup of the Collaboration together with the Project manager constructed three resources for inclusion in the guidelines to accompany the diagnostic instrument for FASD in Australia. The “Information on FASD Diagnostic Assessment for Parents and Carers” describes what is involved in getting a diagnosis for FASD, what documentation or information is useful to provide to the health professionals during the assessment process, what happens after the assessments, why diagnosis is important, an explanation of informed consent, and contact details for FASD support groups in Australia. An “Australian FASD Diagnostic Assessment Consent Form” was also developed. The third resource was “Information for clinicians: Understanding the issues that patients and their parents or carers may experience during the FASD diagnostic assessment”. These issues included listening to the concerns raised by the parents or carers; explaining the assessment process and medical terminology; and recommendations for how to speak to a person undergoing diagnostic assessment for FASD, and their parents or carers.

## Discussion

**Figure 1:**
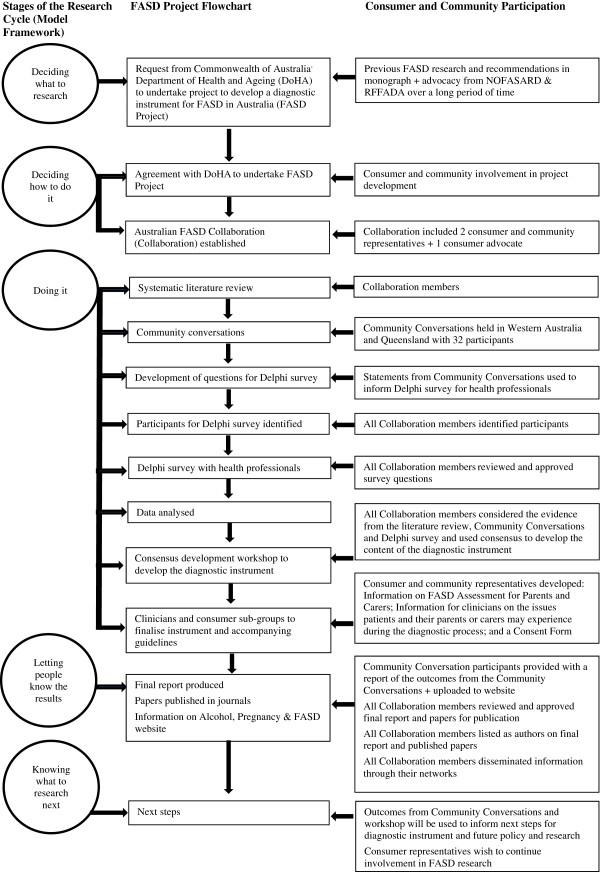
Consumer and community participation in the FASD Project.

In this paper, we have described the involvement of consumers and community members in the development of a diagnostic instrument for FASD in Australia. The process is summarised in Figure 1. Findings highlight the varying views of consumer representatives and researchers and the need for planning and participation throughout the research cycle. Participation by consumers and the community was considered important and essential, and integral to the success of the FASD Project.

The results of the FASD Project Collaboration evaluation survey with respect to consumer and community participation, concur with the outcomes from the questionnaire for consumer and community representatives and researchers in the Alcohol and Pregnancy Project [28] and with views expressed by other consumers involved with research at the Telethon Institute [16]. Participants in all projects agreed that consumer and community participation is essential to good research practice, makes research relevant to the community and can give researchers a real passion and sense of relevance to their work. One factor contributing to the quality of social process is the composition of the group [32]. The Collaboration was multidisciplinary with consumer representatives, clinicians, epidemiologists, researchers and policy makers. The representatives from NOFASARD and RFFADA are engaged consumers who meet the Aslin and Brown criteria of “one who is occupied, focused and expresses a high level of commitment to an issue” [33]. The inclusion of a consumer advocate and two consumer representatives on the Collaboration was also consistent with the values expressed in McKenzie and Hanley [16] and the Patient and Public Involvement Policy of the National Institute for Health and Care Excellence in the UK [34].

The challenge of ensuring that the personal narrative or visceral personal experiences [32] do not interfere with the ability of the consumer and community representatives to consider the evidence and make decisions based on a combination of the evidence and what is most relevant to patients and their families, can be addressed by involving more than one consumer and community representative on a project reference or steering group. Representatives should also be adequately briefed on the project, membership of the group and expectations of each member prior to agreeing to join the group. The antithesis to having consumer and community personal narratives is where researchers may not consider the views of consumers and the community, patients and their families as relevant to their work [16]. In interviews with researchers, patients and health professionals in the UK and the Netherlands, some interviewees thought that knowledge of patients might be useful, but it was hardly worth the trouble and one researcher commented that “*patients should not interfere in processes of which they know nothing about*” [35]. Another UK survey of university-based researchers [36] highlighted the need for greater understanding of the realities of public involvement, the need for education and training, and time to adjust to this new way of working. Findings from the FASD Project reinforced the view that consumers have a role in grounding research and ensuring researchers consider what would work best for affected children and their families.

The processes used in the FASD Project are compatible with the eight principles of successful consumer involvement in National Health Service (NHS) research [37] and used in research by Boote et al. [20] and Payne et al. [28]. The roles of each member of the Collaboration, including consumers and community members, were established prior to the research commencing and each member was acknowledged as bringing different skills, knowledge and experience to the group. Funding, including honoraria for the Collaboration consumer representatives and Community Conversation participants, was allocated for consumer and community participation in the FASD Project. All members of the Collaboration were involved in decisions about recruitment and were actively engaged in the conduct of the research. Consumer participation at all levels has been described in the final report to the Australian Government Department of Health and Ageing; and a Community Conversation report circulated to all participants and available from the Telethon Institute Alcohol, Pregnancy and FASD website [38]. The usefulness of consumer and community involvement in the FASD Project was not diluted by a lack of resourcing, failure to embed consumer involvement in strategic research objectives, funding and unclear responsibility for implementing consumer involvement among key stakeholders as can be seen in some health and medical research in Australia [39]. Further evidence of the increasing role played by consumer and community members is described in the 2011 Telethon Institute Annual Report [40] with the inclusion of a community member on grant review panels, funding for a Consumer and Community Participation Unit, researcher training workshops and Community Conversations.

The FASD Project garnered the views of both consumers with a distinct knowledge of FASD (two consumer representative members of the Collaboration) and the broader community who may not have any, or limited knowledge about alcohol use in pregnancy and FASD (Community Conversations). The Community Conversations allowed women to discuss what is of concern to them with respect to what information health professionals provide about alcohol and pregnancy, how they discuss the issue and what information they as patients would be prepared to disclose. This is in agreement with the views expressed in “Mother knows best: Developing a consumer led, evidence informed, research agenda for maternity care” [41] that sometimes researchers are disease focused whereas aspects of care are important to the majority of women. The key finding from the Community Conversations with respect to health professionals advising women about alcohol use during pregnancy support and extend the findings from other alcohol and pregnancy research. Peadon et al. found that women expected health professionals to ask and advise them about alcohol and pregnancy [42,43]. France et al. indicated that some health professionals were making an assumption that women knew to minimise alcohol consumption during pregnancy [44]. This is consistent with the findings of Cheyne et al. [41] who stated that health professionals often assume that they fully understand patient’s point of view and concerns and that additional efforts to identify these are unnecessary.

## Conclusions

The successful involvement of consumers and the community in the FASD Project can be attributed to the planning and continued involvement throughout the project, funding for their participation, including representation on the Collaboration and for the Community Conversations, and an understanding of the various contributions by each member to the Collaboration.

The participation of consumers in the Collaboration and the Community Conversations, were successful processes for consumer and community input into research. The willingness of these women to provide practical and insightful responses made a significant contribution to the project and will inform planning for future prevention, education, advocacy and research programs relating to alcohol, pregnancy and FASD in Australia. In keeping with the principles in the Statement and research cycle in the Model Framework, the FASD Project demonstrated the “how’s” and benefits of consumer and community participation and the importance of consumers and the community in health and medical research projects. The involvement of consumers and the community in the FASD Project and the willingness by members of the Collaboration to support and enable it were considered a model example of how active consumer and community participation in research can be achieved.

## Competing interests

The authors declare that they have no competing interests.

## Authors’ contributions

All authors were part of the FASD Project to develop the diagnostic instrument for FASD in Australia. AMcK and HMJ developed and organised the Community Conversations. AMcK facilitated the Community Conversations. AMcK, SM and ER formed the consumer subgroup with HMJ acting as facilitator. HMJ developed the on-line evaluation questionnaire and analysed the data. HMJ drafted the manuscript and all authors edited the manuscript. All authors read and approved the final version of the manuscript.
